# Supplementary material for: Activation of neural lineage networks and ARHGEF2 in enzalutamide-resistant and neuroendocrine prostate cancer and association with patient outcomes
Source: Commun Med (Lond). 2022 Sep 21;2:118. doi: 10.1038/s43856-022-00182-9 (PMC9492734; doi:10.1038/s43856-022-00182-9)
Supplement: Supplementary file 1 — Supplementary information [file 43856_2022_182_MOESM1_ESM.pdf]

Supplementary Information

Activation of neural lineage networks and ARHGEF2 in enzalutamide-resistant and neuroendocrine prostate cancer and association with patient outcomes

Shu Ning[1], Jingge Zhao[1,2], Alan P Lombard[1], Leandro S D'Abronzo[1], Amy R Leslie[1], Masuda Sharifi[1], Wei Lou[1], Chengfei Liu[1,3], Joy C Yang[1], Christopher P Evans[1,3], Eva Corey[4], Hongwu Chen[3,5], Aiming Yu[3,5], Paramita M Ghosh[1,3,5,6], Allen C Gao[1,3,6]

[1]Department of Urologic Surgery, University of California Davis, Sacramento, CA, USA

[2] Present address: Department of Urology, West China Hospital, Sichuan University, Sichuan, China

[3]UC Davis Comprehensive Cancer Center, University of California Davis, Sacramento, CA, USA

[4]Department of Urology, University of Washington, Seattle, WA, USA

[5]Department of Biochemistry and Molecular Medicine, University of California Davis, Sacramento, CA, USA

[6]VA Northern California Health Care System, Sacramento, CA, USA

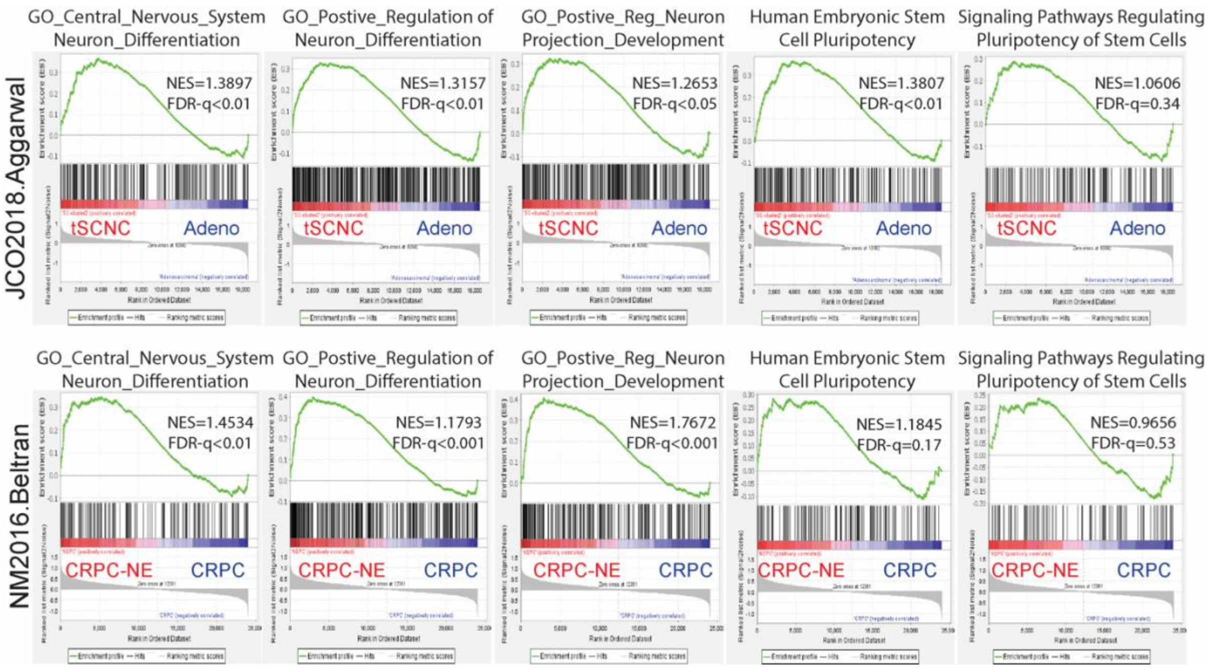

Supplementary Figure 1.

Transcriptional analysis implicates a neural lineage feature in small cell/NEPC patients. GSEA pathway analysis using the GO and Pathcards gene sets are shown. The enrichment plots with |NES|>1 and FDR q-value < 0.05 were shown that five neural related pathways were activated in tSCNC of Aggarwal cohort compared with prostate adenocarcinoma, and CRPC-NE patients of Beltran cohorts.

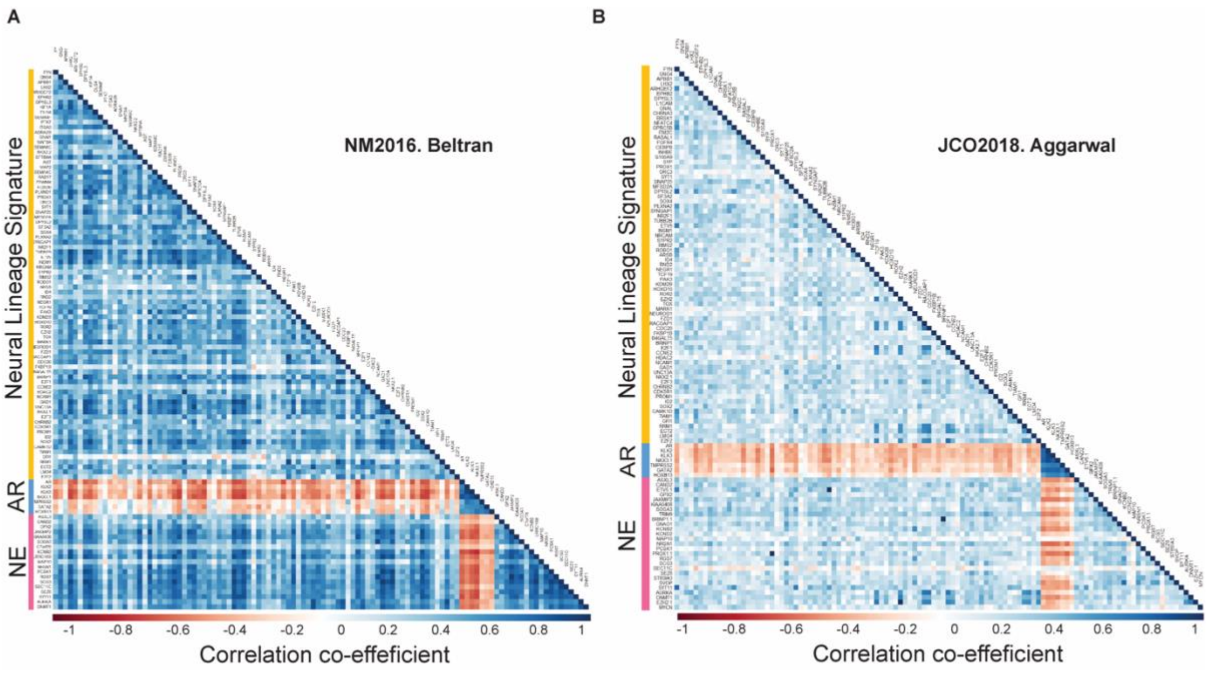

Supplementary Figure 2

Heatmap depicting correlation of the gene expression of neural lineage signature, AR and NE markers in Beltran **(a)** and Aggarwal **(b)** patient samples. NLS genes were correlated with classical NE markers and negative correlates with AR markers. The correlation coefficients were present in colour blue to red to indicate high to low correlation.

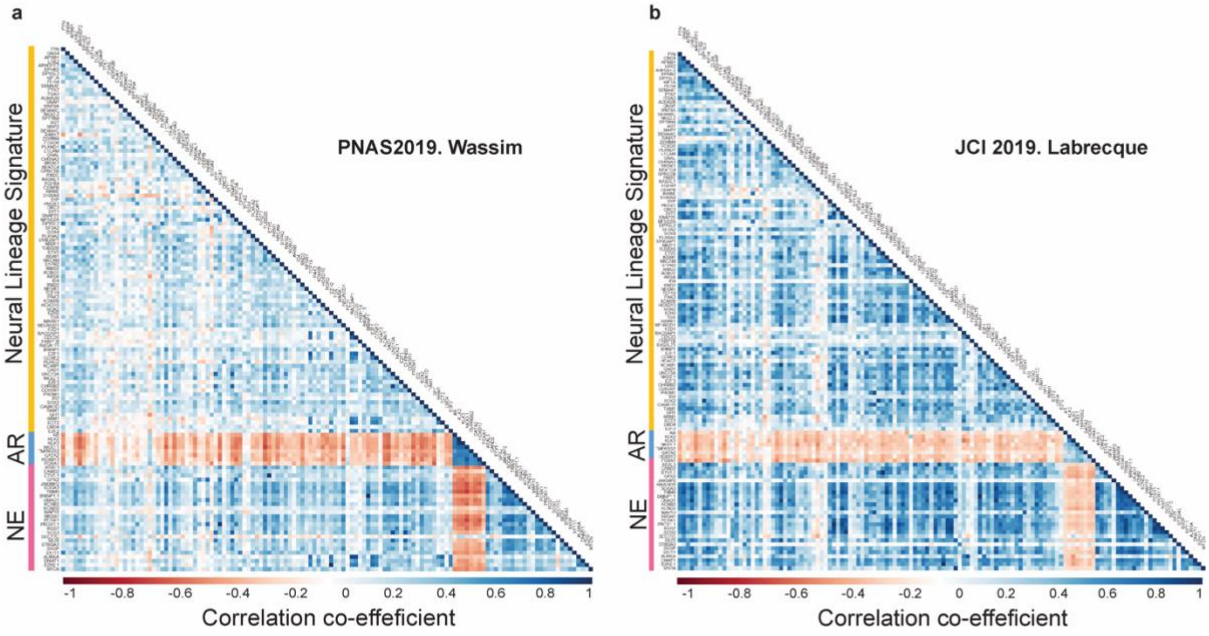

Supplementary Figure 3.

Heatmap depicting correlation of expression level of neural lineage signature, AR and classical NE markers in Wassim **(a)** and Labrecque databases **(b)**. NLS genes were positively correlated with classical NE markers, while negatively correlates with AR markers. High correlation was coloured in blue, and negative correlation was coloured in red.

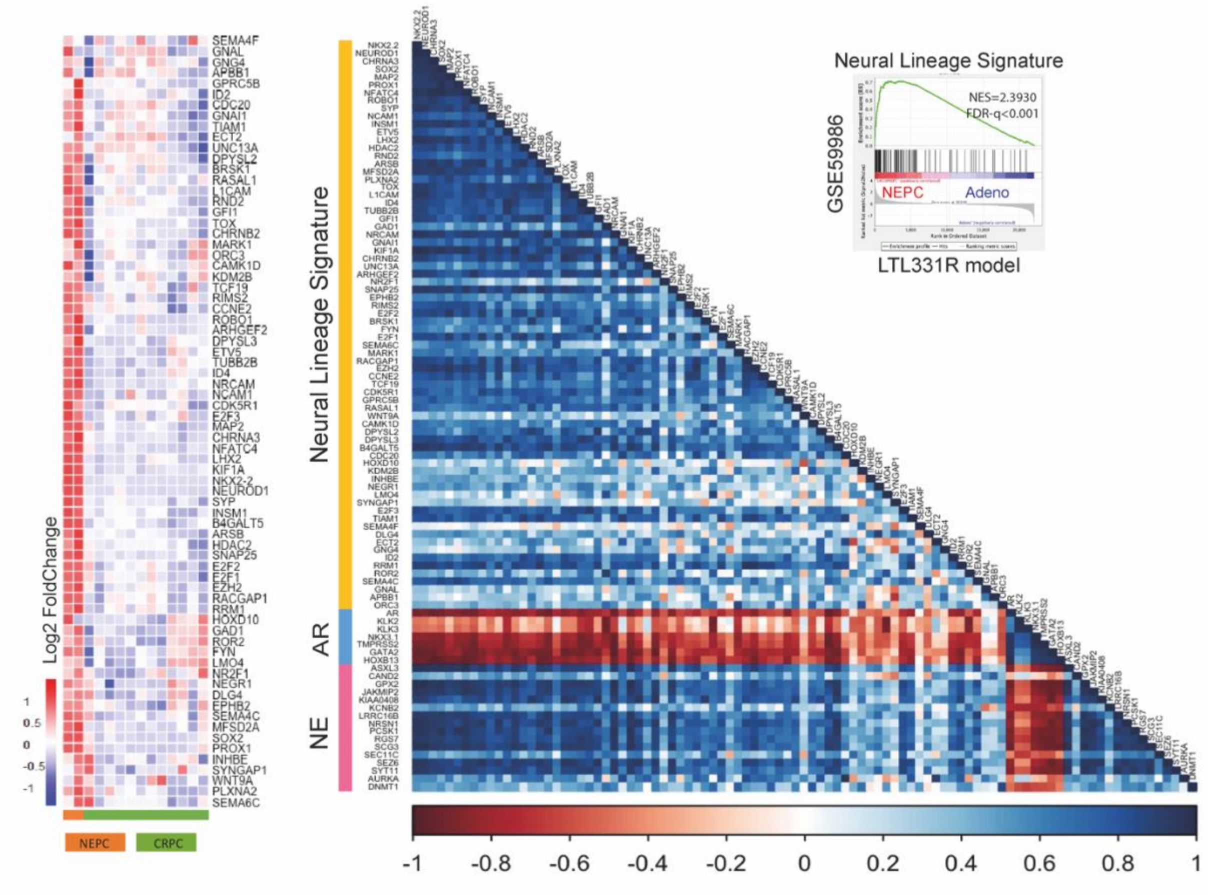

Supplementary Figure 4

Hierarchical clustering heatmap demonstrates the upregulated genes in neural lineage signature (Left). Heatmap depicting correlation of the gene expression of neural lineage, AR and NE markers in LTL331R NEPC model. NLS genes were positively correlated with NE markers (blue), but negatively correlates with AR and its target genes (red). GSEA enrichment plots showing the enrichment of NLS genes in TLT331R model NEPC groups compared to adenocarcinoma groups).

Pathway Genesets	Source	NES	FDR-q
GO_Neural crest cell migration	GSEA	-2.1699	0.0010
WP_Neural crest cell migration in cancer	GSEA	-1.4036	0.0998
GO_Positive regulation of neuron differentiation	GSEA	-1.6856	0.0010
GO_Positive regulation of neuron projection development	GSEA	-1.5413	0.0010
WP_Neural crest differentiation	GSEA	-1.8002	0.0010
GO_Central nervous system neuron differentiation	GSEA	-1.4065	0.0206
LEE_Neural crest stem cell_DN	GSEA	-1.4573	0.0187
GO_Neural precursor cell proliferation	GSEA	-1.5984	0.0051
GO_Neural crest cell differentiation	GSEA	-1.9548	0.0010
GO_Neuron cellular homeostasis	GSEA	-1.3007	0.1546
GO_Positive regulation of neural precursor cell proliferation	GSEA	-1.2977	0.1389
Bhattacharya_Embryonic _Stem_Cell	GSEA/PMID15070671	-1.0346	0.4048
Boquest_Stem_Cell _Up	GSEA/PMID15635089	-1.5605	0.0000
Neural Stem Cell Differentiation Pathways and Lineage-specific Markers	Pathcards	-1.7172	0.0068
Human Embryonic Stem Cell Pluripotency	Pathcards	-1.6255	0.0000
Signaling pathways regulating pluripotency of stem cells	Pathcards	-1.1616	0.2099
GO_Regulation of stem cell population maintenance	GSEA Broad Institute	-1.5032	0.0508
GO_Regulation of stem cell differentiation	GSEA Broad Institute	-0.9134	0.6185
Ramalho_Stemness _Up	GSEA/PMID12228720	0.8378	0.8104
GO_Regulation of neuron projection regeneration	GSEA	-1.1043	0.3177
GO_Regulation of neuron projection development	GSEA	-1.8358	0.0010
GO_Regulation of neuron differentiation	GSEA	-1.8963	0.0010
GO_Axon	GSEA	-1.8983	0.0010

Supplementary Table 1.
GSEA pathway enrichment analysis demonstrates neural development related pathways were enriched in C4-2B MDVR cell line. Normalized enrichment score (NES) was summarized with corresponding FDR-q values showing pathway activation in C4-2B MDVR cells.
