# Supplementary material for: Activation of neural lineage networks and ARHGEF2 in enzalutamide-resistant and neuroendocrine prostate cancer and association with patient outcomes
Source: Commun Med (Lond). 2022 Sep 21;2:118. doi: 10.1038/s43856-022-00182-9 (PMC9492734; doi:10.1038/s43856-022-00182-9)
Supplement: Supplementary file 4 — Description of Additional Supplementary Files [file 43856_2022_182_MOESM4_ESM.pdf]

Description of Additional Supplementary Files

File Name: Supplementary Data 1

Description: Neural lineage signature 95 gene list

File Name: Supplementary Data 2

Description: All data supporting this study and uncropped blot images
