# Supplementary material for: Activation of neural lineage networks and ARHGEF2 in enzalutamide-resistant and neuroendocrine prostate cancer and association with patient outcomes
Source: Commun Med (Lond). 2022 Sep 21;2:118. doi: 10.1038/s43856-022-00182-9 (PMC9492734; doi:10.1038/s43856-022-00182-9)
Supplement: Supplementary file 6 — Reporting Summary [file 43856_2022_182_MOESM6_ESM.pdf]

nature research

Corresponding author(s): Allen C Gao

Last updated by author(s): 07-20-2022

Reporting Summary

Nature Research wishes to improve the reproducibility of the work that we publish. This form provides structure for consistency and transparency in reporting. For further information on Nature Research policies, see our Editorial Policies and the Editorial Policy Checklist.

Statistics

For all statistical analyses, confirm that the following items are present in the figure legend, table legend, main text, or Methods section.

n/a	Confirmed	
☐	☒	The exact sample size (*n*) for each experimental group/condition, given as a discrete number and unit of measurement
☐	☒	A statement on whether measurements were taken from distinct samples or whether the same sample was measured repeatedly
☐	☒	The statistical test(s) used AND whether they are one- or two-sided<br>*Only common tests should be described solely by name; describe more complex techniques in the Methods section.*
☐	☒	A description of all covariates tested
☐	☒	A description of any assumptions or corrections, such as tests of normality and adjustment for multiple comparisons
☐	☒	A full description of the statistical parameters including central tendency (e.g. means) or other basic estimates (e.g. regression coefficient) AND variation (e.g. standard deviation) or associated estimates of uncertainty (e.g. confidence intervals)
☒	☐	For null hypothesis testing, the test statistic (e.g. *F*, *t*, *r*) with confidence intervals, effect sizes, degrees of freedom and *P* value noted<br>*Give P values as exact values whenever suitable.*
☒	☐	For Bayesian analysis, information on the choice of priors and Markov chain Monte Carlo settings
☒	☐	For hierarchical and complex designs, identification of the appropriate level for tests and full reporting of outcomes
☐	☒	Estimates of effect sizes (e.g. Cohen's *d*, Pearson's *r*), indicating how they were calculated

Our web collection on statistics for biologists contains articles on many of the points above.

Software and code

Policy information about availability of computer code

Data collection	no software used.
Data analysis	Analyses were conducted using the R studio (version 2021.09.1, https://www.r-project.org/). The heatmaps were generated using 'pheatmap' (version 1.0.12). The correlation plots were generated using R pakage 'corrplot' (version 0.92). The Kaplan-Meier Survival analyses were generated using R package 'survival' (version 3.2-13).

For manuscripts utilizing custom algorithms or software that are central to the research but not yet described in published literature, software must be made available to editors and reviewers. We strongly encourage code deposition in a community repository (e.g. GitHub). See the Nature Research guidelines for submitting code & software for further information.

Data

Policy information about availability of data

All manuscripts must include a data availability statement. This statement should provide the following information, where applicable:

- Accession codes, unique identifiers, or web links for publicly available datasets
- A list of figures that have associated raw data
- A description of any restrictions on data availability

Tumor sample information and corresponding clinical characteristics from Beltran cohort castration resistant neuroendocrine prostate cancer 13, Stand Up 2 Cancer/Prostate Cancer Foundation-funded West Coast Prostate Cancer Dream Team 20, and Abida-Wassim cohort metastatic castration-resistant prostate cancer 21 were downloaded from cBioPortal for Cancer Genomics (www.cbioportal.org). Whole-genome RNA sequencing for treatment-resistant metastatic castration prostate cancer 19 was obtained from NCBI's Gene Expression Omnibus (GEO) using the accession number GSE126078. Gene expression and clinical information from The Cancer Genome Atlas Research Network 22 were downloaded from the cBioPortal for Cancer Genomics (https://www.cbioportal.org/study/summary?cancer_study_id=prad_tcga_pub). Transcriptomes and corresponding clinical information from Memorial Sloan-Kettering Cancer Center (MSKCC) were downloaded

from cBioPortal (https://www.cbioportal.org/study/summary?id=prad_mskcc) and NCBI GEO under accession GSE21032. Microarray data of LTL331R NEPC tumor model was downloaded from NCBI GEO under accession number GSE59986. Gene expression in 22RV1, LNCaP95, MSKCC-EF1 and H660 were obtained using GEO accession number GSE154576 (https://www.ncbi.nlm.nih.gov/geo/query/acc.cgi?acc=GSE154575).
The RNA sequence data in the present study has been deposit to NCBI's Gene Expression Omnibus (GEO) using the accession number GSE64143. All data are available from the authors upon request. Source data are provided with this paper in Supplementary Data 1.

Field-specific reporting

Please select the one below that is the best fit for your research. If you are not sure, read the appropriate sections before making your selection.

☒ Life sciences ☐ Behavioural & social sciences ☐ Ecological, evolutionary & environmental sciences

For a reference copy of the document with all sections, see nature.com/documents/nr-reporting-summary-flat.pdf

Life sciences study design

All studies must disclose on these points even when the disclosure is negative.

Sample size	We use all the available data from the four patient study (NM2016.Beltran, JCO2018.Aggarwal, JCI2019.Labrecque, PNAS2019.Wassim)
Data exclusions	We did not exclude the sample.
Replication	None.
Randomization	None.
Blinding	None.

Reporting for specific materials, systems and methods

We require information from authors about some types of materials, experimental systems and methods used in many studies. Here, indicate whether each material, system or method listed is relevant to your study. If you are not sure if a list item applies to your research, read the appropriate section before selecting a response.

Materials & experimental systems

n/a	Involved in the study	
☐	☒	Antibodies
☐	☒	Eukaryotic cell lines
☒	☐	Palaeontology and archaeology
☐	☒	Animals and other organisms
☒	☐	Human research participants
☐	☒	Clinical data
☒	☐	Dual use research of concern

Methods

n/a	Involved in the study	
☒	☐	ChIP-seq
☒	☐	Flow cytometry
☒	☐	MRI-based neuroimaging

Antibodies

Antibodies used	AR 376 (441)(Santa Cruz Biotechnology, Santa Cruz, CA)<br>GEF-H1 (55B6) Rabbit mAb 377 from Cell Signaling Technology (Catalog#4076)<br>CHGA from Santa Cruz Biotechnology 378 (Catalog#393941)<br>NSE from Santa Cruz Biotechnology (Catalog#9116)<br>GAPDH (14C10) From Cell Signaling Technology (Catalog#2118)
Validation	Data provided in the manuscript.

Eukaryotic cell lines

Policy information about cell lines

Cell line source(s)	Prostate cancer C4-2B cells obtained from the American type Culture Collection (ATCC).<br>Enzalutamide resistant C4-2B MDVR cells were generated from C4-2B cells by maintaining in 20µM enzalutamide for over 10 months.<br>H660 cells were obtained from ATCC.
Authentication	C4-2B parental and MDVR, and H660 cell line were used after acquired from original source.

Mycoplasma contamination	All cell lines have been routinely tested mycoplasma free by PCR and authenticated by short tandem repeat (STR) method.
Commonly misidentified lines (See ICLAC register)	No commonly misidentified cell lines are used in this study

Animals and other organisms

Policy information about studies involving animals; ARRIVE guidelines recommended for reporting animal research

Laboratory animals	C.B17/lcrHsd-Prkdc-SCID mice (ENVIGO), mouse, male, 6-8weeks.
Wild animals	This study did not involve any wild animals.
Field-collected samples	This study did not involve samples collected from the filed.
Ethics oversight	All animal studies were performed and approved by the Institutional Animal Care and Use Committee of UC Davis.

Note that full information on the approval of the study protocol must also be provided in the manuscript.

Clinical data

Policy information about clinical studies

All manuscripts should comply with the ICMJE guidelines for publication of clinical research and a completed CONSORT checklist must be included with all submissions.

Clinical trial registration	*Provide the trial registration number from ClinicalTrials.gov or an equivalent agency.*
Study protocol	*Note where the full trial protocol can be accessed OR if not available, explain why.*
Data collection	*Describe the settings and locales of data collection, noting the time periods of recruitment and data collection.*
Outcomes	*Describe how you pre-defined primary and secondary outcome measures and how you assessed these measures.*
